# Ascarosides from helminths pack a punch against allergy

**DOI:** 10.1073/pnas.2202250119

**Published:** 2022-03-30

**Authors:** Rick M. Maizels

**Affiliations:** ^a^Wellcome Centre for Integrative Parasitology, Institute of Infection, Immunity and Inflammation, University of Glasgow, Glasgow G12 8TA, United Kingdom

In every encounter between different species, critical recognition processes inform and direct the response on each side. In settings such as parasitism, the interaction is exploited by an invading organism to manipulate its host and facilitate its establishment. Frequently, the signals dispatched by parasites have evolved from physiological pathways governing their own development and reproduction, adapted for a novel function environment of the host. There is no better example than found in an exciting new report, in PNAS (1), that pheromone-like ascaroside signaling molecules, released from an intestinal nematode parasite, act to block the host immune response mode that would be most damaging to the worm.

Nematode roundworms, together with cestode tapeworms and trematode flukes, collectively termed helminths, are near-ubiquitous parasites in low-resource countries (2). Immunity to helminths is dependent on the type 2 arm of the immune response, orchestrated by Th2 helper lymphocytes and their cytokines (such as IL-4 and IL-5), driving IgE antibodies and innate effector cells such as macrophages and eosinophils (3). The same set of players mediate allergic reactions, suggesting that allergy may be the consequence of a poorly regulated immune system with an overzealous type 2 response.

Globally, there is an inverse correlation between helminth prevalence and inflammatory disorders such as allergy and autoimmune disease, suggesting that helminths can dampen type 2 immunity across the board (4). Where deliberate helminth infection of humans has been trialed, some patients experience alleviation of symptoms, but this therapeutic strategy faces numerous challenges (5), and increasing emphasis is placed on laboratory models to identify pathways of parasite immunomodulation and the molecular players involved (6).

Accordingly, a fascinating palette of molecular mediators from helminths are now being defined (7). In the case of allergy, one example is the “ARI” (alarmin release inhibitor), a 20-kDa protein in the excretory/secretory (ES) products of the intestinal nematode *Heligmosomoides polygyrus* that blocks release of IL-33, a central player in both allergy and helminth immunity (8). However, nearly all helminth modulatory molecules so far described are secreted proteins; the demonstration that ascarosides may act to similar effect now shines the spotlight on small molecules.

Ascarosides were first identified as players in larval development and mate attraction in the free-living nematode *Caenorhabditis elegans* (9). These small glycolipids comprise an ascarylose (didesoxymannose) sugar coupled to short (three- to six-carbon) aliphatic side chains (10). Other organisms recognizing ascarosides include nematode-predatory fungi which respond by forming traps (11). Notably, plants exposed to nematode ascarosides activate innate immune responses, enhancing resistance not only to parasites but to microbial pathogens introduced at the same time (12).

Shinoda et al. (1) provide a compelling and exciting window into how helminths can manipulate the immune system of their host; while ascarosides had been shown to activate plant immunity, this is a demonstration of mammalian responses, with an attenuating effect.

*Nippostrongylus brasiliensis* (*Nb*) is, like *H. polygyrus*, a rodent nematode widely used as a laboratory model; an earlier study found *Nb* ES to block allergy even when the macromolecular fraction was heat or proteinase treated (13). Surmising that the active factor might therefore be a small molecule bound to a protein carrier, Shinoda et al. (1) isolated the <3-kDa fraction from *Nb* and tested it for suppression of allergic reactivity. Their results have opened an intriguing dimension in parasitology and the immunology of allergy.

The authors (1) used a laboratory model of airway allergy, sensitizing mice by intraperitoneal injection of ovalbumin in the Th2-promoting adjuvant alum, before ovalbumin challenge in the airways. Inflammatory immune cells (in particular, eosinophils) infiltrate the lungs and alveolar fluid, while goblet cells in the airway epithelium express high levels of mucin; these reactions were almost entirely ablated if the small-molecule fraction of *Nb* ES (smES) was added to the ovalbumin/alum mixture at the time of priming. In addition, the authors obtained similar results with a “real-world” allergen from house dust mites, in which sensitization is entirely in the airways, and no alum adjuvant is required.

**Fig. 1.**
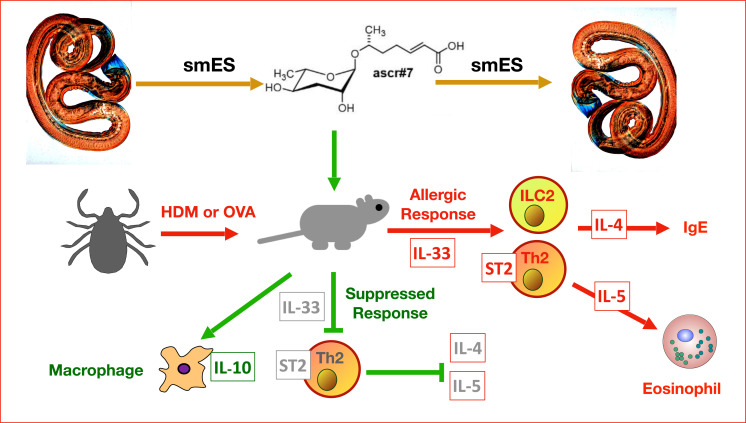
*Nb* parasites release pheromones such as ascr#7, found in smES materials; these act on other members of the same species (*Top Right*) while also modulating the host type 2 immune response and alarmin cytokines such as IL-33. The type 2 response in allergy to house dust mite (HDM) or ovalbumin (OVA) activates type 2 helper T cells (Th2) and innate lymphoid cells (ILC2) which express the IL-33 receptor (ST2) and release cytokines such as IL-4 (that drives IgE production) and IL-5 (which induces eosinophils). If ascr#7 is coadministered to mice with allergen, expression of IL-33 and ST2 is abated, the type 2 response is suppressed, and macrophage expression of the suppressive cytokine IL-10 is enhanced.

Next, the authors (1) fractionated the smES by high-performance liquid chromatography, and tested eight separate fractions in vivo. While several fractions showed inhibition (and one showed exacerbation), only two showed significant blockade of eosinophilia, and these were the only two to contain one or more of four previously characterized ascarosides, designated ascr#1, ascr#3, ascr#7, and ascr#10. When synthetic versions of these four molecules were tested, three (including ascr#1) had protective effects, but only one showed strong suppression of cellular infiltration into the lungs; this was ascr#7 (Fig. 1).

What effects do ascarosides exert on the mammalian immune system? To address this question, immune cell populations (expressing the panhematopoietic marker CD45) from allergic and ascr#7-treated mice were isolated and analyzed by single-cell sequencing; ascr#7 administration caused a significant reduction in Th2 cells (identified by the presence of the transcription factor *Gata3*) expressing the IL-33 receptor ST2 (encoded by *Il1rl1*). Lung T cells challenged in vitro with ovalbumin also mounted weaker IL-4 and IL-15 responses if derived from ascr#7-exposed mice. Delving further, the inhibition of ST2 extended systemically to the spleen, and affected innate cell populations such as lung ILC2s which were diminished in number (Fig. 1).

Interference with IL-33 signaling then focused attention on the airway epithelium, which is the natural source of IL-33 in the initial stages of allergic reactivity, sparking ILC2 activation in the lung (14). IL-33 expression in epithelial cells, bearing the EpCam marker but negative for CD45, was elevated in allergen-sensitized mice, but not if the animals had also received ascr#7. Moreover, the macrophage population was modulated by ascr#7, with recipient mice showing an increase in expression of IL-10, an effective antiinflammatory cytokine with known effects in airway allergy.

As *Nb* is only one of a vast range of nematode species, many of which are known to synthesize ascarosides (10), the authors (1) cast a wider net, examining other parasitic species across the nematode phylogenetic tree: All were found to release these molecules. In particular, ascr#1 and ascr#7 are found not only in species more closely related to *C. elegans* (in this case, the human hookworm *Ancylostoma ceylanicum* and the mouse species *H. polygyrus*) but also in the distantly related Trichinellids (the pork worm *Trichinella spiralis* and the pig whipworm *Trichuris suis*); ascr#1 is secreted in vitro by each species, and ascr#7 is secreted by all but *T. spiralis*. The ascarosides are, however, restricted to nematodes, raising the question of whether the flatworms (cestodes and trematodes) express analogous pheromones that might also impact the host immune system.

Strikingly, the ability of ascr#7 to inhibit IL-33 expression is not unique among secreted products of helminths. As well as *Hp-*ARI mentioned above, a second protein from *H. polygyrus* (*Hp-*BARI) binds and blocks the receptor for IL-33, ST2 (15), while extracellular vesicles released from the same worm down-regulate ST2 at the messenger RNA level (16). The concerted targeting of the IL-33/ST2 pathway by different nematodes is likely to reflect the essential role it plays in the protective immune response to helminth infection. Interestingly, this may not be the only tool in the ascaroside box: There are hints that ascr#7 may expand the host immunoregulatory network, as Tregs appeared to increase at the expense of Th2 cells, and IL-10–expressing macrophages were found.

Taken together, Intriguingly, a physiological product with evolutionary origins as a mating pheromone has been repurposed by parasites to dampen host immunity. Quite possibly, the ascarosides now fulfill both functions, rendering them obligate for successful mating of the worm. In this case, they would represent an indispensable nematode-specific molecular signature, akin to the microbial pathogen-associated molecular patterns such as bacterial cell wall lipopolysaccharide (LPS) or flagellin (17).

As with any discovery, questions soon outnumber answers. The studies of Shinoda et al. (1) were conducted in vivo, so it is not known whether immune cells in vitro can be modulated by ascarosides, and whether the effects on Th2 cells and eosinophils, for example, are direct responses to the nematode molecules, or rely on interactions with a third-party cell type. In this regard, it will be important to determine the receptors in mammalian cells that sense the presence of ascarosides, and whether these are, as in *C. elegans*, members of the G protein–coupled receptor family. The authors’ characterization of differential activity from very closely related molecular structures will greatly assist in researching the identity and behavior of host cell receptors involved in this system.

Stepping back, it is curious that the mammalian immune system has not learned, as have plants (12), to recognize ascarosides as danger signals (like bacterial LPS), but instead respond by down-regulation. Here the downstream signals resulting from ascaroside treatment would be instructive, for example, if they involve the TGF-β and/or insulin pathways as in *C. elegans*. Another possibility is that the immune system selects a tolerant response to the nanomole quantities the authors (1) used in their experiments, with high doses perhaps exerting an opposite effect.

Finally, the authors (1) suggest that, in light of the reservations for live helminth therapy of inflammatory disorders, ascarosides may provide a fertile alternative, avoiding the perils of introducing live parasites into the human body. As well as being available in synthetic form, they also have the merit, as small molecules, of not evoking a host antibody response that might neutralize their function if repeatedly administered to patients. Clearly, many new opportunities are now opening to understand, and take advantage of, how helminths shape their host environment.
